# Herpes Zoster in Persons Living with HIV-1 Infection: Viremia and Immunological Defects Are Strong Risk Factors in the Era of Combination Antiretroviral Therapy

**DOI:** 10.3389/fpubh.2018.00070

**Published:** 2018-03-12

**Authors:** Nathaniel B. Erdmann, Heather A. Prentice, Anju Bansal, Howard W. Wiener, Greer Burkholder, Sadeep Shrestha, Jianming Tang

**Affiliations:** ^1^Department of Medicine, University of Alabama at Birmingham, Birmingham, AL, United States; ^2^Department of Epidemiology, University of Alabama at Birmingham, Birmingham, AL, United States

**Keywords:** herpes zoster, human immunodeficiency virus type 1, incident rate, epidemiology, correlation

## Abstract

In a cohort of 4,225 persons living with human immunodeficiency virus type 1 (HIV-1) infection (PLWH) enrolled at a southeastern US clinic, the overall rate of incident herpes zoster (HZ) was 101 per 10,000 person-years (PY) between January 1999 and 2017, which nearly quadruples the rate reported for the general US population. In the same cohort, the median age of HZ diagnosis was 39.5 years [interquartile range (IQR) 31.5–49.2] in African American (AA) and 39.1 years (IQR 34.9–45.2) in European American (EA) PLWH, with the highest incidence seen in PLWH who were over 50 years old (144 and 93 per 10,000 PY in AA and EA, respectively, *P* = 0.18), showing no bias between men (100 per 10,000 PY) and women (101 per 10,000 PY). In multivariable models that were applicable to 245 HZ cases and 3,713 controls, age, nadir CD4^+^ T-cell (CD4) count, plasma viral load (VL), and duration of combination antiretroviral therapy were independent correlates of incident HZ (adjusted *P* ≤ 0.006 for all). Regardless of other factors, viremic PLWH (VL > 50 copies/mL) was at the highest risk of HZ [adjusted odds ratio (OR) > 3.0, *P* < 0.0001]. PLWH with a nadir CD4 count of ≥500 cells/μL showed a relatively low risk (adjusted OR = 0.48, *P* = 0.003). By contrast, similar risk estimates were observed with three advancing age groups (30–39, 40–49, and ≥50) when compared with age <30 (adjusted OR = 1.86–2.17, *P* ≤ 0.010). These findings indicate that efforts for HZ diagnosis and prophylaxis should target viremic PLWH who are over 30 years old and with CD4 count <500 cells/μL.

## Introduction

Herpes zoster (HZ) is a debilitating, but readily diagnosed disease that results from reactivated varicella-zoster virus (VZV) infection. An episode of HZ may last for weeks to months and occasionally leads to complications like postherpetic neuralgia (PHN). According to estimates from the United States Centers for Disease Control and Prevention (CDC), about one million American adults develop HZ each year, while one-third of Americans develop HZ in their lifetime (1). Notably, a vast majority of these HZ cases are seen in the elderly population, as HZ rates increase substantially after age 50 in the US general population (2). This age bias has prompted the CDC to recommend active HZ vaccination for all persons over 60 years without contraindications (1).

Apart from advanced age, other known risk factors for HZ include gender, race, stress, statin use, and various states of immunosuppression seen in malignancy, transplantation, and human immunodeficiency virus type 1 (HIV-1) infection (2–6). In persons living with HIV-1 infection (PLWH), HZ has been well recognized as a common complication since early in the epidemic, particularly in individuals who are well below the age of onset typical in the general population. Several cohort studies in Africa, Europe, and North America have provided strong epidemiological evidence that combination antiretroviral therapy (cART) can reduce HZ, but its incidence, recurrence, and complications (particularly PHN) remain more common in PLWH than in the general population, which may reflect enduring problems with suboptimal adherence to cART, partial immune reconstitution (as measured by CD4^+^ T-cell counts), lingering stress, or accelerated/premature aging (5, 7–14).

To identify correlates and trends of HZ in the context of cART, we have examined a well-defined, southeastern US clinical cohort of PLWH who had longitudinal data between January 1999 and 2017. Our findings suggest that efforts for HZ prophylaxis may need to focus on PLWH who are over 30 years old and with CD4 count of <500 cells/μL. These PLWH, especially those who have detectable HIV-1 viral load (VL), should be of primary interest in follow-up studies that aim to uncover correlates of immune dysregulation or to assess the benefits of effective vaccination. Identifying these correlates and determining the underlying mechanisms for the risk of HZ may also be useful in understanding non-AIDS comorbidities in PLWH.

## Subjects and Methods

### Study Population

**Figure 1:**
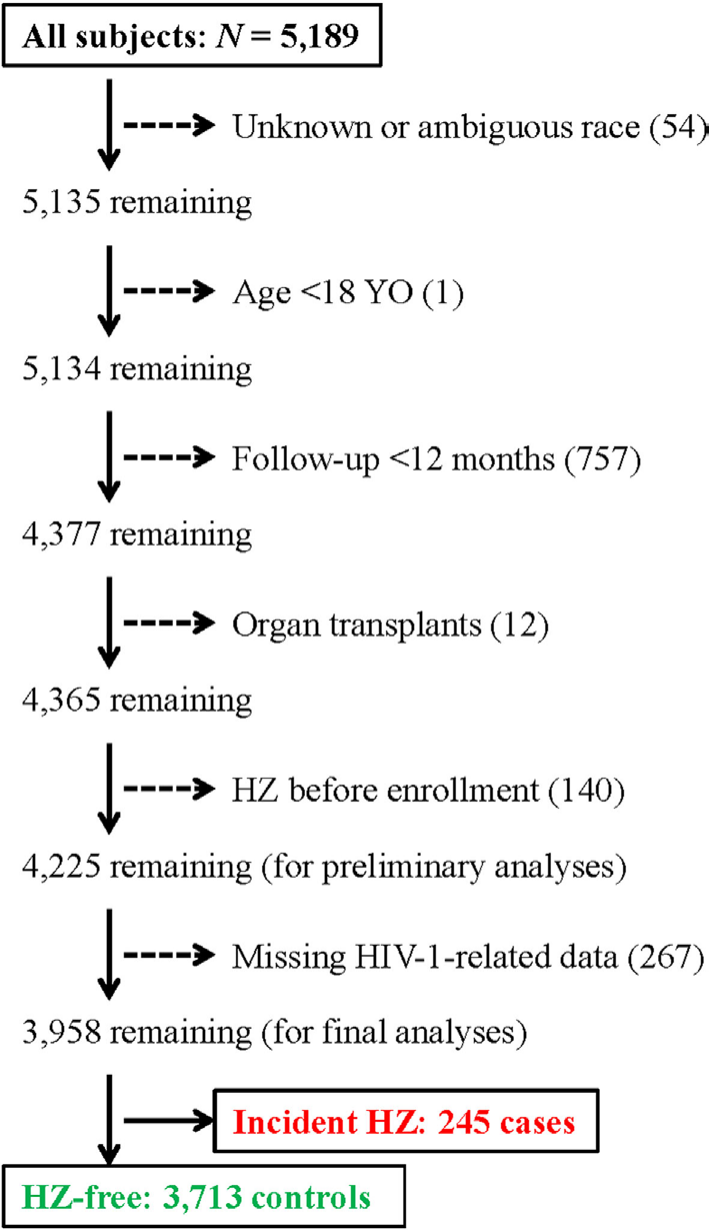
A flow chart for the selection of 245 incident herpes zoster (HZ) cases and 3,713 control subjects for this study. A total of 4,225 subjects are included in initial analyses.

From a well-established clinical cohort of PLWH enrolled at a local clinic in Birmingham, Alabama (The 1917 Clinic) (15), we selected 4,225 subjects (out of a total of 5,189) for the initial assessment of incident HZ between January 1999 and 2017 (Figure 1). Excluded subjects included (i) 54 with unknown or ambiguous race (self-reported), (ii) one who was less than 18 years old; (iii) 757 HZ-free subjects who has <12 months of follow-up after the first clinical visit (enrollment); (iv) 12 who had organ transplants (receiving immunosuppressive therapy); and (v) 140 who reported HZ before enrollment (prevalent HZ cases). For multivariable modeling, we further focused on 3,958 PLWH, including 245 incident cases and 3,713 control subjects, who had multiple measurements of HIV-1 VL and CD4^+^ T-cell (CD4) counts to allow a full evaluation of virological and immunological correlates (267 PLWH were excluded because of missing data). All subjects provided written informed consent for participation in the prospective, multifaceted cohort study, and research protocols pertinent to this substudy were approved by an Institutional Review Board at the University of Alabama at Birmingham.

### Patient Data for Various Analyses

The following parameters from this southeastern US cohort of PLWH were retrieved from a central repository: (i) demographics (sex, baseline age, race/ethnicity); (ii) risk factors for HIV-1 acquisition (sexual and injection drug use); (iii) the duration of cART-free infection and the duration of cART; (iv) virological failure after cART (detection of viral blips), (v) systemic coinfections (e.g., hepatitis C virus genotype and plasma HCV RNA), (vi) three comorbidities (asthma, diabetes, and psoriasis) that were relatively common in the cohort; (vii) nadir CD4 count before HZ diagnosis in the last 12 months of follow-up for the control subjects, (viii) absolute CD4 count and CD4 percentage in the two visits immediately before HZ diagnosis or the last two visits in control subjects, (ix) HIV-1 VL in the three visits immediately before HZ diagnosis or the last three visits in control subjects (to determine the highest VL during these intervals), and (x) calendar dates of enrollment and diagnosis of incident HZ. In addition, prescribed medication and medical charts were used to verify incident HZ diagnosis. The incidence rate of HZ was calculated as cases per 1,000 person-years (PY) of active follow-up (after enrollment). Cases per calendar year were also evaluated for trends between January 2000 and December 2016 (excluding two tail ends with sparse data).

### Descriptive Statistics

Baseline demographics and characteristics for the study cohort were captured in descriptive statistics (Tables 1 and 2), which included (i) counts (before and after stratifications) and percentages/frequencies for various observations, (ii) mean and standard deviations (SD) of the mean for quantitative measures that conform to a normal distribution (e.g., age) and (iii) median and interquartile range (IQR) for quantitative measures that deviated from a normal distribution (e.g., age at HZ diagnosis and VL). The rates of HZ occurrence were expressed as mean and 95% confidence interval (CI) per 10,000 PY of follow-up. For consistency with earlier reports, baseline age was categorized (mostly per decade), so were nadir CD4 count, CD4 percentages, and plasma VL (<50 copies/mL was considered as undetectable). For factors that were available for association analyses, their pairwise correlation (extent of collinearity) was evaluated using the Spearman method. Trend of HZ incidence over time was assessed using linear regression (for slope and *P*-value).

**Table 1:** Overall characteristics of the study cohort stratified by the status of herpes zoster (HZ).

Baseline characteristics	HZ-free subjects^a^ (*N* = 3,973)	Incident HZ cases^a^ (*N* = 252)	*P*-value^b^
Age at enrollment (mean ± SD)	39.5 ± 11.1	40.0 ± 9.8	>0.40
Age category at enrollment			0.029
18–29 years	907 (22.8)	39 (15.5)	
30–39 years	1,243 (31.3)	93 (36.9)	
40–49 years	1,096 (27.6)	77 (30.6)	
≥50 years	727 (18.3)	43 (17.1)	
Sex			>0.80
Women	923 (23.2)	60 (23.8)	
Men	3,050 (76.8)	192 (76.2)	
Race/ethnicity			>0.80
African American (AA)	2,387 (60.1)	149 (59.1)	
European American (EA)	1,510 (38.0)	99 (39.3)	
Others	76 (1.9)	4 (1.6)	
HIV-1 risk factor			0.105
Men having sex with men (MSM)	2,207 (55.6)	129 (51.2)	
Heterosexual	1,644 (41.4)	119 (47.2)	
Others (including injection drug use)	122 (3.1)	4 (1.6)	
HCV infection status			0.201
No HCV infection	3,666 (92.3)	225 (89.3)	
Infection with HCV genotypes 1a/1b	250 (6.3)	23 (9.1)	
Infection with other HCV genotypes	57 (1.4)	4 (1.6)	
Comorbidity 1: asthma	419 (10.6)	25 (9.9)	>0.75
Comorbidity 2: diabetes (predominant type 2)	440 (11.1)	21 (8.3)	0.176
Comorbidity 3: psoriasis	69 (1.7)	3 (1.2)	>0.50
Nadir CD4 count^c^ (missing in 32 subjects)			<0.0001
<200 cells/μL	1,906 (48.4)	159 (63.1)	
200–500 cells/μL	1,305 (33.1)	70 (27.8)	
>500 cells/μL	730 (18.5)	23 (9.1)	
Nadir CD4 percentage^c^ (missing in 42 subjects)			<0.0001
<15%	1,643 (41.8)	142 (56.4)	
15–29%	1,573 (40.0)	90 (35.7)	
30–40%	559 (14.2)	17 (6.8)	
>40%	156 (4.0)	3 (1.2)	
Duration of cART (before the end of follow-up)			<0.0001
Untreated	98 (2.5)	13 (5.2)	
Up to 24 months	616 (15.5)	84 (33.3)	
>24 months	3,259 (82.0)	155 (61.5)	
Plasma viral load (VL)^c^ (missing in 235 subjects)			<0.0001
<50 copies/mL	2,288 (61.1)	56 (22.9)	
50–4,245^d^ copies/mL	595 (15.9)	54 (22.0)	
>4,245^d^ copies/mL	862 (23.0)	135 (55.1)	

*^a^Numbers are shown as *N* and column percentage (%), unless specified otherwise*.

*^b^Statistical significance is shown for reach parameter*.

*^c^As measured in the last 12 months of follow-up (between January 1999 and 2017). Nadir CD4^+^ T-cell (CD4) count and highest viral load are analyzed*.

*^d^For the study period, 4,245 copies/mL is the upper limit of the interquartile range*.

**Table 2:** Independent correlates of incident herpes zoster (HZ), as defined by a reduced multivariable models.

Factors in the final model	*N*(%)^b^	Adjusted OR^c^	95% CI	*P*-value
**Age at enrollment^a^**				
18–29 years	876 (22.1)	ref.	–	
30–39 years	1,237 (31.3)	2.03	1.35–3.06	<0.001
40–49 years	1,109 (28.0)	2.17	1.42–3.32	<0.001
≥50 years	736 (18.6)	1.86	1.16–2.97	0.010
**Nadir CD4^+^ T-cell count^a^**				
<200 cells/μL	1,943 (49.1)	ref.	–	
200–500 cells/μL	1,291 (32.6)	0.73	0.53–1.00	0.050
>500 cells/μL	724 (18.3)	0.48	0.30–0.79	0.003
**Duration of ART^a^**				
Untreated	88 (2.2)	ref.	–	
Up to 24 months	647 (16.4)	1.06	0.52–2.13	>0.85
>24 months	3,223 (81.4)	0.42	0.21–0.84	0.014
**Plasma viral load^a^**				<0.0001
<50	2,337 (59.0)	ref.	–	
50–4,245	642 (16.2)	3.07	2.07–4.55	<0.0001
>4,245	979 (24.7)	4.99	3.55–7.02	<0.0001

*^a^As listed in Table 1; adjusted *P* ≤ 0.006 for each of these four parameters (the basis for further testing within each parameter)*.

*^b^Overall N = 3,958, when 267 subjects are excluded because of missing data for HIV-1 viral and/or CD4 count*.

*^c^The referent group (ref.) under each parameter is not necessarily the largest (the norm)*.

### Central Hypothesis and Analytical Procedures

To identify independent correlates of incident HZ, this study tested an overarching hypothesis that factors that are unequivocally associated with HZ incidence could inform health-care decisions. The potential (either reported or hypothesized) predictors were first screened in univariable models that evaluated one factor at a time through case–control comparisons. Individual parameters with a nominal *P*-value of <0.05 accompanied by a false discovery rate (*q-*value) of <0.05 were accepted as provisional correlates and then further tested in multivariable models (omnibus tests). All factors with adjusted *P* < 0.05 were retained in a reduced multivariable model, and their relative impact on HZ incidence was gauged by adjusted odds ratio (OR) and 95% CI. For factors (demographics and HIV-risk groups) that did not change over time, Kaplan–Meier curves and Cox proportional hazard models were also applied to evaluate their potential impact on time from enrollment to HZ onset. These analytical procedures were conducted using software packages in SAS, version 9.2 (The SAS Institute, Cary, NC, USA).

## Results

### Rates of HZ Incidence in the Study Cohort

As of January 2017, 4,225 subjects from our study cohort met the criteria for initial analyses (Figure 1). These included 252 incident HZ cases and 3,973 control subjects, with a total of 25,106 PY of follow-up since January 1999. Overall, the rate of incident HZ was 101 per 10,000 PY (95% CI 88–113 per 10,000 PY), which was almost identical to the rate (103 per 10,000 PY) previously reported for immunocompromised subjects (including PLWH) seen in 2000 and 2001 (2). When stratified by sex and race, the HZ rates were equivalent for men (101 per 10,000 PY) and women (99 per 10,000 PY) (*P* > 0.50) but differed somewhat between AA (111 per 10,000 PY) and European American (EA) (88 per 10,000 PY) (*P* = 0.067).

### Age at HZ Occurrence

**Figure 2:**
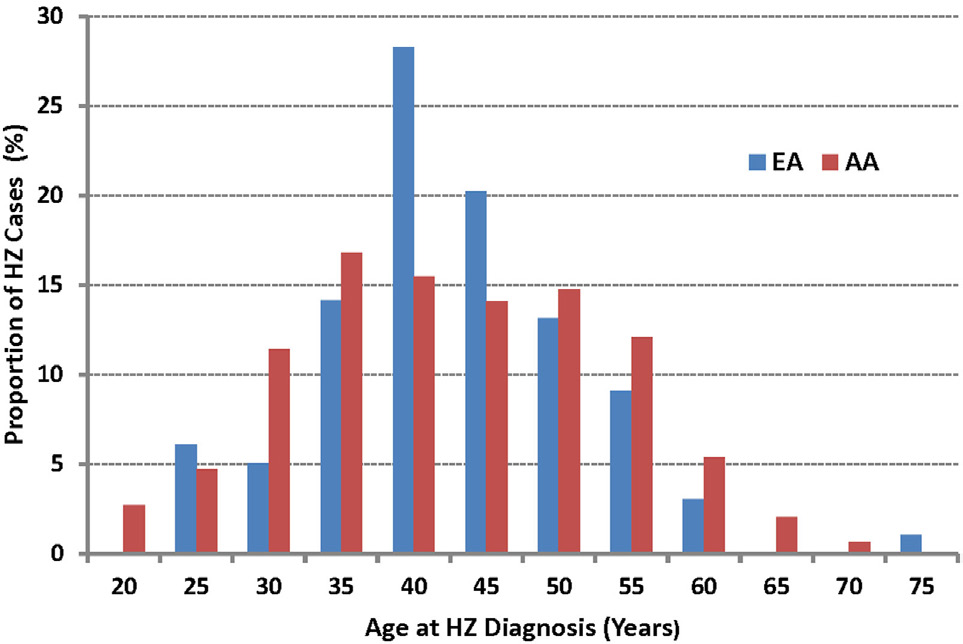
The age of onset for incident herpes zoster (HZ) cases as observed in African American (AA) and European American (EA) persons living with HIV-1 infection. The median age at HZ diagnosis is 39.5 years in AA and 39.1 years in EA (*P* > 0.50).

The median age of HZ diagnosis was similar for AA (39.5 years) and EA (39.1 years) (IQR 31.5–49.2 and 34.9–45.2, respectively), both substantially younger than reported in the general US population (2). The actual age distribution differed between the two primary races found in this cohort (*P* = 0.036 by likelihood ratio test) (Figure 2), but the numbers within each age stratum were too small to facilitate further evaluation.

### Potential Predictors of HZ Occurrence

In a direct, step-by-step comparison, HZ cases and control subjects were highly comparable in terms of sex, race, and HIV-1-risk factors (*P* > 0.10 for all) (Table 1). Comorbidities including asthma and diabetes (predominantly type 2) were found in about 10% of the cohort, while psoriasis was relatively rare (<2%) in both HZ cases and controls (*P* > 0.15). On the other hand, the distribution of four age categories (18–29, 30–39, 40–49, and ≥50 years) differed somewhat between HZ cases and the control subjects (*P* = 0.029, *q* = 0.072). In contrast, cases and controls showed stark differences in the distribution of all HIV-1-related outcomes of interest, including three categories of a nadir CD4 count (<200, 200–500, and >500 cells/μL), four categories of nadir CD4 percentage (<15, 15–29, 30–40, and >40%), three categories of cART duration (untreated, up to 24 months of cART, and >24 months of cART), as well as three plasma VL groups (<50, 50–4,245, and >4,245 copies/mL) (*P* < 0.0001 for all comparisons).

### Assessment of Collinearity (Spearman Correlation) among Patient Characteristics

For the majority of patient parameters that were included in the analyses, pairwise correlation analyses revealed weak or no collinearity (Spearman |ρ| < 0.40) (data not shown). The only exceptions were (i) men versus men having sex with men (ρ = 0.61); (ii) nadir CD4 < 200 cells/μL versus CD4 percentage <15% (ρ = 0.63); (iii) nadir CD4 > 500 cells/μL versus nadir CD4 percentage 30–40% (ρ = 0.44). These pairwise relationships were statistically significant (*P* < 0.001 for all comparisons) but not strong enough to confound a joint assessment of their relative roles in a multivariable model.

### Independent Predictors of HZ Occurrence

In a reduced multivariable model applicable to 3,958 subjects with no missing data (245 HZ cases and 3,713 controls), age, nadir CD4 count, plasma VL, and duration of cART remained as independent correlates of HZ incidence (adjusted *P* ≤ 0.006 for all) (Table 2). Viremic subjects (VL > 50 copies/mL) were at the highest risk of HZ (adjusted OR > 3.0, *P* < 0.0001), regardless of other factors. Subjects with a nadir CD4 count of ≥500 cells/μL showed a relatively low risk (adjusted OR = 0.48, *P* = 0.003). Meanwhile, similar risk estimates were seen with three advancing age groups (30–39, 40–49, and ≥50) when compared with age <30 (adjusted OR = 1.86–2.17, *P* ≤ 0.010). These associations were robust in alternative analyses that were restricted to 2,369 AA and 1,458 EA subjects (data not shown).

### Gradual Decline in HZ Occurrence

**Figure 3:**
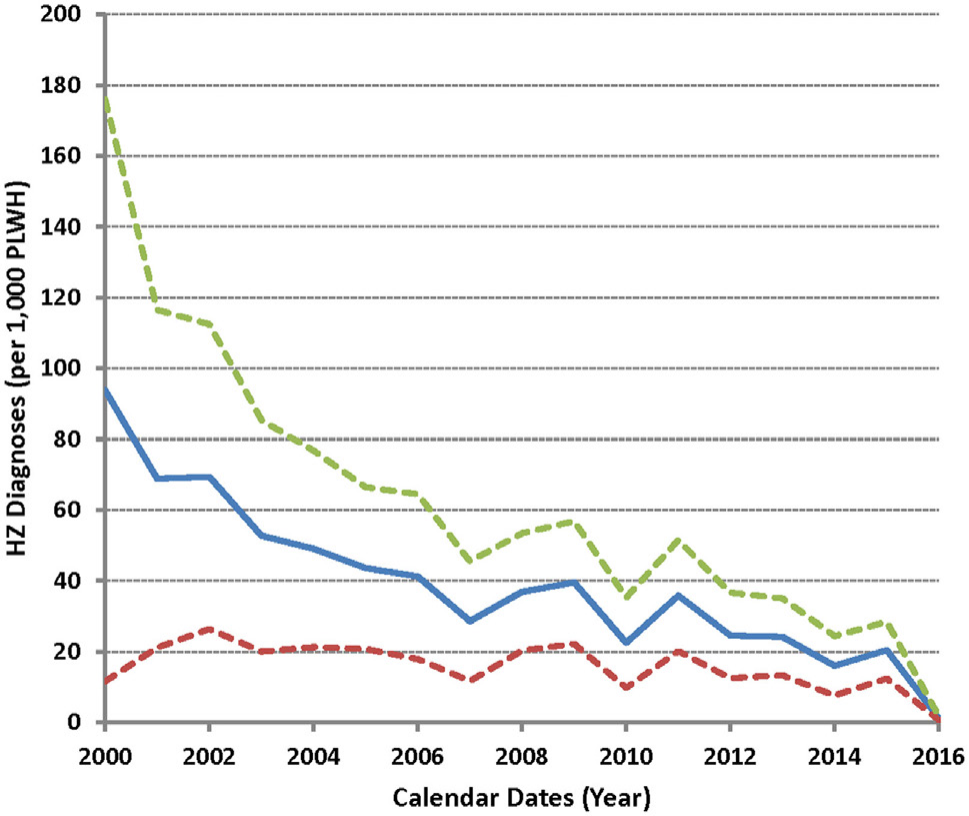
Incidence of herpes zoster (HZ) as observed between 2000 and 2016 in the study cohort. Solid line indicates the average HZ diagnoses (per 1,000 subjects) over time (in yearly intervals), while dotted lines correspond to the 95% confidence intervals.

As few HZ cases (two each) were seen during 1999 and in January 2017, a test for trend was applied to annual data between 2000 and 2016 (Figure 3). A successive decrease in HZ frequency was apparent, with an estimated annual reduction of 4 cases per 1,000 PLWH (*P* = 1.6 × 10^−7^ for a linear fit). The annual drop was more dramatic between 2000 and 2007 (8 per 1,000 PLWH) as compared to the later periods (3 per 1,000 PLWH) (*P* = 0.006 between the two intervals).

### Low Frequency of Recurrent HZ

A subset of 14 HZ cases reported a second episode of HZ that ranged from 23 months to 12 years after the first episode, while a single patient reported a third episode that was 24 months after the second. These recurrent cases did not appear to cluster by HZ severity or any specific patient characteristics or demographics.

### Confirmatory Findings from Kaplan–Meier Curves and Cox Proportional Hazard Models

**Figure 4:**
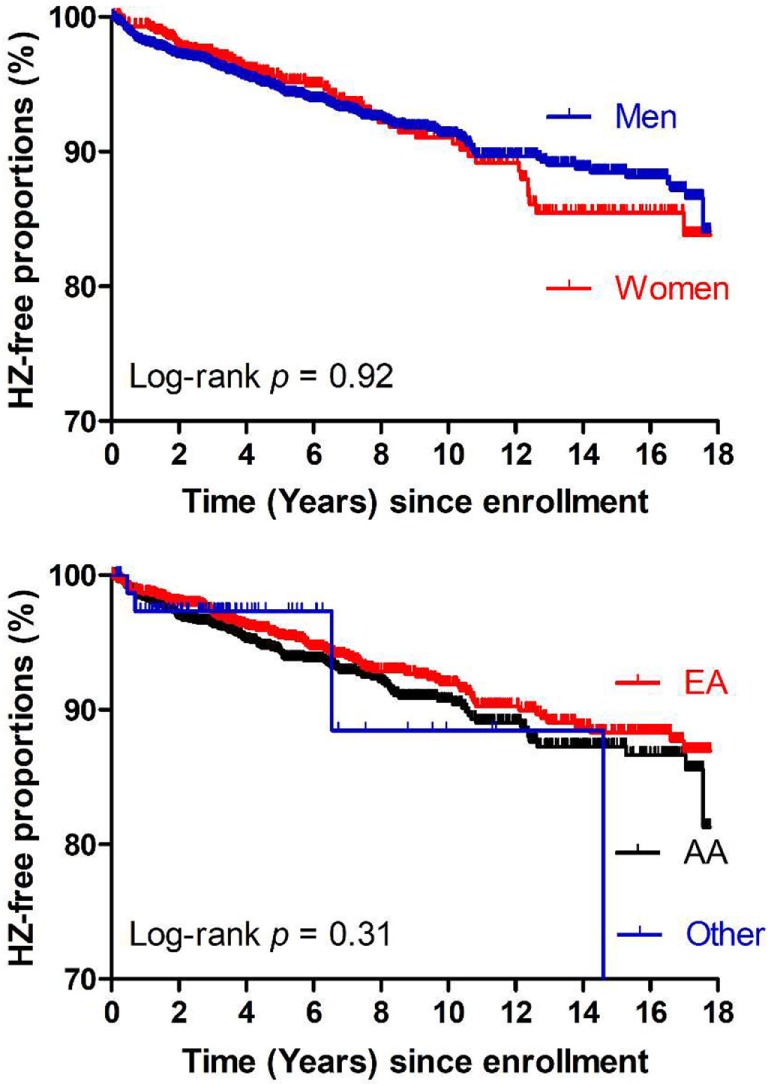
Kaplan–Meier curves showing time from study enrollment (January 1999) to events of herpes zoster (HZ) in the study population (*N* = 4,225) stratified by sex (top panel) or race (bottom panel). No clear differences are seen between men and women or among three ethnic groups (AA, African American; EA, European American), being consistent with logistic regression models (Table 1).

Consistent with results from logistic regression models (Table 1), Kaplan–Meier curves and Cox proportional hazards models revealed that neither sex nor ethnicity influenced HZ occurrence in our study cohort (log-rank *P* = 0.92 and 0.31, respectively) (Figure 4), suggesting again that key findings summarized in Table 2 should be generalizable (regardless of sex or ethnicity).

## Discussion

Results from our observational study here clearly indicate that HZ occurrence remains relatively high for PLWH in the era of cART, regardless of race, sex, HIV-1 risk factor, HCV coinfection, or several comorbidities. In our relatively young, clinical cohort, access to cART already exceeds the goal (90%) set by the World Health Organization, with fewer than 2.6% of the subjects not on therapy. Yet, the overall rate of incident HZ (101 per 10,000 PY) nearly quadruples the rate previously reported for the general US population (30 per 10,000 PY) (16) or approaches the rate (130 per 10,000 PY) for elderly (≥60 years old) Americans who did not receive HZ vaccination (17). Efforts to dissect the underlying biology and to provide effective interventions are still relevant to the management of contemporary PLWH.

The median age (39 years old) at HZ occurrence in our cohort is younger than that of the uninfected population (over 50 years) (1, 2). PLWH over 30 years old are already at an elevated risk for HZ compared to uninfected, elderly individuals. This age bias was particularly evident in individuals with a nadir CD4 count below 200 cells/μL. Patients with CD4 nadir between 200 and 500 cells/μL only had a slight advantage (adjusted OR = 0.73) as compared with CD4 nadir below 200 cells/μL, suggesting that any preventive interventions need to reach a broad base of the PLWH population in terms of age and immune reconstitution. This can be challenging when other comorbidities in aging PLWH compete for priorities and resources.

As two major risk factors for HZ occurrence in PLWH, nadir CD4 counts below 200 cells/μL and detectable viremia reflect three well-anticipated problems with (i) lack of early HIV-1 diagnosis and linkage to care, (ii) partial immune reconstitution after cART initiation, and (iii) suboptimal cART adherence in some patients. At least one study has demonstrated that VZV-specific, cell-mediated immunity is impaired in adult PLWH with a history of AIDS and low CD4 counts (18), which implies the importance of T-helper cells to maintaining VZV-specific, cytotoxic T-lymphocytes (19). The extent to which these subjects respond to new, subunit VZV vaccines (20, 21) remains a question, but alleviation of contraindications associated with the current, live attenuated VZV vaccine should encourage clinical trials in high-risk PLWH groups with low CD4 counts.

In previous studies of PLWH in the USA, one report based on data from January 1997 to December 2001 revealed that over one-third of HZ patients had complications (including PHN) despite relatively young age and that severely immunocompromised patients may develop HZ shortly after cART initiation (7). A second study of PLWH noted a lower-than-expected HZ rate between 2002 and 2009 (8). By our assessment, the trend for an evolving HZ landscape is discernible, especially around 2007 (Figure 3), but the number of HZ cases per calendar year is too small to allow a definitive conclusion. By contrast, the rates of incident HZ in the general US population appeared to be steady between 1992 and 2002 (22).

The majority of HZ incidence in our study population occurs within the first 2 years of cART, which is highly consistent with findings from the Women’s Interagency HIV Study (11). Immune recovery can be highly heterogeneous within this time window (23), but the relatively low rate of recurrent HZ is perhaps an encouraging sign that VZV-specific immunity is boosted even in the setting of HIV infection. A gradual and seemingly biphasic decline in HZ occurrence between 1999 and 2017 may further reflect the improved efficacy and various clinical advantages of new cART regimens.

A key advantage in our study cohort is the inclusion of diverse ethnic groups and HIV risk factors, as well as adequate representation of both sexes (Table 1). Both case–control comparisons and Kaplan–Meier curves firmly demonstrate that race and sex have no major role in HZ occurrence, indicating that findings derived from our study should be generalizable.

A potential limitation in our study is that the identification of HZ cases relies heavily on retrospective medical records. Clinical diagnosis and documentation can fluctuate over time, which may translate to under-reporting in the medical record and favor negative (null) findings. Another potential concern is uncertainty with vaccination history. To date, recommendations on clinical implementation of the live HZ vaccine in PLWH have been ambivalent, although expert opinion supports vaccinating patients who have recovered CD4 counts (to avoid complications) (24–27). In our own cohort, HZ vaccination has a limited coverage (reducing uptake), but some patients do receive HZ vaccine outside of the clinic setting and choose to not file a report. These circumstances can be further complicated by the difficulty in mounting protective immune responses in viremic individuals with significant immune dysregulation. When new, safe vaccine strategies become available, a new focus should be to optimize the timing of vaccination for maximum benefit.

In summary, our work here demonstrates a high HZ incidence even in relatively young PLWH under broad implementation of cART. Elevation in HZ occurrence is undoubtedly magnified in viremic patients with abnormal CD4 count. Establishing a safe and effective protocol for the clinical management of these high-risk PLWH may require some close attention to the timing of interventional strategies (28).

## Ethics Statement

This study was carried out in accordance with the recommendations of human experimentation guidelines set forth by the Department of Health and Human Services, United States of America, with written informed consent in accordance with the Declaration of Helsinki. The research protocol was approved by an Institutional Review Board at the University of Alabama at Birmingham.

## Author Contributions

JT, NE, AB, and SS designed the study. GR supervised the data management team. HW and HP analyzed the data. NE and JT drafted the manuscript, while all coauthors edited various draft versions before approving the submission for publication.

## Conflict of Interest Statement

The authors declare that the research was conducted in the absence of any commercial or financial relationships that could be construed as a potential conflict of interest. The reviewer LZ and the handling editor declared their shared affiliation.
